# Discrimination of the commercial Korean native chicken population using microsatellite markers

**DOI:** 10.1186/s40781-015-0044-6

**Published:** 2015-02-05

**Authors:** Nu Ri Choi, Dong Won Seo, Slim Ben Jemaa, Hasina Sultana, Kang Nyeong Heo, Cheorun Jo, Jun Heon Lee

**Affiliations:** Department of Animal Science and Biotechnology, College of Agriculture and Life Sciences, Chungnam National University, Daejeon, 305-764 Republic of Korea; INRA-Tunisie, Laboratoire des Productions Animales et Fourragères, Rue Hédi Karray, 2049 Ariana Tunisia; Poultry Science Division, National Institute of Animal Science, RDA, Cheonan, 331-801 Republic of Korea; Department of Agricultural Biotechnology, Center for Food and Bioconvergence, and Research Institute for Agriculture and Life Science, Seoul National University, Seoul, 151-921 Republic of Korea

**Keywords:** Discrimination, Diversity, Microsatellite, Korean native chicken, Woorimatdag

## Abstract

**Background:**

Korean native chicken (KNC) is a well-known breed due to its superior meat taste. This breed, however, owing to a low growth rate, has a high market price. In order to overcome this disadvantage, the National Institute of Animal Science (NIAS) in Korea developed a commercial KNC breed, named Woorimatdag version 2 (WM2), an upgraded version of the Woorimatdag (WM1) breed and the WM2 was created by crossing the KNC with meat type breeds. This study aims to discriminate between WM2 and other chicken breeds using microsatellite (MS) markers.

**Methods:**

A total of 302 individuals from eight Korean chicken populations were examined. The genetic diversity and population structure analysis were investigated using Cervus, API-CALC, STRUCTURE, PowerMarker programs.

**Results:**

Based on heterozygosity and polymorphic information content (PIC) values, 30 MS markers were initially selected from 150 markers. The identified average number of alleles (Na), expected heterozygosity, and PIC values for the WM2 samples were 7.17, 0.741, and 0.682, respectively. Additionally, the paternity of individuals was assigned with a success rate of greater than 99% using 12 markers, the best minimum number of markers. The 12 selected markers contained heterozygosity and PIC values above 0.7 and probability of identity values around zero. Using these markers, the determined probability of identity (*PI)*, *PI*_*half-sibs*_, and *PI*_*sibs*_ values were 3.23E-33, 5.03E-22, and 8.61E-08, respectively.

**Conclusions:**

WM2 is well differentiated with respect to other chicken breeds based on estimated genetic distances. The results presented here will contribute to the identification of commercial WM2 chicken in the market.

**Electronic supplementary material:**

The online version of this article (doi:10.1186/s40781-015-0044-6) contains supplementary material, which is available to authorized users.

## Background

Recently, chicken meat consumption in Korea has rapidly increased to 12 kg per capita due to consumer preferences for healthy white muscle meat [1]. In comparison to red meat, chicken meat is considered a healthier option because of lower fat, cholesterol, and iron levels [2]. Presently, approximately 90% of the Korean poultry industry contains imported chicken breeding stocks. The breeds that existed before the Korean War (1950–1953), unfortunately, are almost all extinct. Since 1992, a Korean native chicken (KNC) conservation project was launched by the National Institute of Animal Science (NIAS) in an attempt to restore local chicken breeds. Recently, five KNC lines and seven others originally imported in the 1960s have been restored [3]. Consumers tend to pay more for the KNCs because of their good taste. The low productivity of the native breeds, however, was disadvantageous for farmers trying to meet feeding and consumption rates. In order to overcome these disadvantages, NIAS developed the Woorimatdag version 1 (WM1) chicken population. WM1 was a commercial, KNC population generated from crossbreeding fast growing native male chickens and good tasting female chickens with increased egg production. WM1 chicken grows faster, reaching the marker weight of 1.8 kg, than the purebred KNC [4]. Moreover, WM1 chickens produce good quality meat with a high oleic acid content, which improves both taste and water holding capacity [4]. Jung et al. [5] reported that WM1 chickens have a significantly higher content of arachidonic acid and meat flavor than commercial broilers (Br). NIAS recently developed Woorimatdag version 2 (WM2) chickens, a modified version of WM1 chickens with increased growth rates.

Traditional methods to identify chicken breeds focused on general appearances such as feather color, shank color, and body type [6]. Because of the process of distribution and market, chicken breeds cannot be effectively classified based on the appearance of meat. Recent advances in molecular biology techniques, however, have provided new opportunities to assess genetic variability at the DNA level [7]. Therefore, many groups have attempted to discriminate breeds using molecular genetic markers. For example, Korean cattle and pork industries developed discrimination and traceability systems using microsatellite (MS) markers [8,9].

MS markers or simple-sequence repeat (SSR) markers, are highly polymorphic, one to six base pair repeats, widely used since they are numerous, randomly distributed in the genome, and show co-dominant inheritance [10,11]. In addition, MS markers were used in the construction of linkage map of quantitative trait locus (QTL) studies [12]. MS markers may be useful in discriminating individuals. The International Society for Animal Genetics (ISAG) has recommended 30 MS markers for breed identification [13].

In a previous study, 97 MS marker variations, including the 30 MS ISAG recommended markers were investigated in 12 chicken populations. While the majority of the 12 population studies were purebred, the commercial chickens were different, products of three- and four-way crosses. Therefore, in this study, the commercial KNC population, WM2 was investigated to discriminate it from the other chicken populations in the market.

## Methods

### Sample collection and DNA extraction

A total of 302 individuals from eight Korean chicken population (187 WM2, 17 WM1, 13 Hanhyup-3 (Hh), 14 Hyunin (Hn), 14 Rhode Island Red (RIR), 15 Cornish Black (CoL), 15 Cornish Red (CoR), 17 Ogye (O) and 10 Br) were examined. Chicken populations care facilities and procedures met or exceeded the standards established by the Committee for Accreditation of Laboratory Animal Care at National Institute of Animal Science (NIAS) in Korea. The study also was conducted in accordance with recommendations described in “The Guide for the Care and Use of Laboratory Animals” published by the institutional Animal Care and Use Committee (IACUC) of NIAS (2012-C-037) in Korea. Genomic DNA was extracted from embryo tissues of WM2 using the PrimePrep™ Genomic DNA isolation kit for tissue (GeNetBio, Korea) and blood samples of other population using the PrimePrem™ Genomic DNA isolation kit for blood. The concentration of DNA samples was measured using NanoDrop 2000C spectrophotometer (Thermo Scientific, USA) and stored at −20°C.

### Microsatellite (MS) marker genotyping

Previously, 150 MS markers were investigated for the discrimination of five purebred KNC lines [14]. From these results, a total of 30 MS markers were initially selected, which have high expected heterozygosity (Hexp) and polymorphic information content (PIC) values for classification of the WM2 and other commercial populations (Table 1). Selected 30 MS markers were distributed on 15 autosomes.

**Table 1:** Primer information for 30 microsatellite markers used in this study*

**Marker**	**Chr.**	**Dye**	**Forward (5′ → 3′)**	**Reverse (5′ → 3′)**	**Allele size (bp)**
ADL0268	1	PET	CTCCACCCCTCTCAGAACTA	CAACTTCCCATCTACCTACT	105-117
MCW0111	1	NED	GCTCCATGTGAAGTGGTTTA	ATGTCCACTTGTCAATGATG	98-112
MCW0145	1	FAM	ACTTTATTCTCCAAATTTGGCT	AAACACAATGGCAACGGAAAC	181-211
**MCW0063**	**2**	**FAM**	**GGCTCCAAAAGCTTGTTCTTAGCT**	**GAAAACCAGTAAAGCTTCTTAC**	**132-150**
**MCW0087**	**2**	**NED**	**ATTTCTGCAGCCAACTTGGAG**	**CTCAGGCAGTTCTCAAGAACA**	**267-283**
**LEI0141**	**2**	**FAM**	**CGCATTTGATGCATAACACATG**	**AAGGCAAACTCAGCTGGAACG**	**220-242**
**MCW0039**	**2**	**VIC**	**CATTGGACTGAGATGTCACTGCAG**	**ACATTTGTCTAATGGTACTGTTAC**	**127-147**
**MCW0264**	**2**	**FAM**	**CTTACTTTTCACGACAGAAGC**	**AGACTGAGTCACACTCGTAAG**	**224-240**
MCW0288	2	FAM	GATCTGCTTCTCTGCCCCATG	GGTACTGTCACCAGAATGAGC	108-122
MCW0127	3	VIC	GAGTTCAGCAGGAATGGGATG	TGCAATAAGAGAAGGTAAGGTC	227-241
MCW0040	3	VIC	ACTCAAAAATGTGGTAGAATATAG	ACCGAAATTGAGCAGAAGTTA	121-145
**ADL0317**	**4**	**FAM**	**AGTTGGTTTCAGCCATCCAT**	**CCCAGAGCACACTGTCACTG**	**178-204**
LEI0094	4	FAM	GATCTCACCAGTATGAGCTGC	TCTCACACTGTAACACAGTGC	254-280
ADL0292	5	FAM	CCAAATCAGGCAAAACTTCT	AAATGGCCTAAGGATGAGGA	110-138
MCW0029	5	VIC	GTGGACACCCATTTGTACCCTATG	CATGCAATTCAGGACCGTGCA	139-189
**ROS0013**	**5**	**NED**	**TGCTGCTCCTGGRAAATTG**	**GAAAAGCCATGGAGGAATCA**	**220-242**
**ADL0159**	**6**	**VIC**	**GCCATTATTTTTCCCTGTGT**	**CTCCCCAAAGTCATTAGCAG**	**107-127**
ROS0019	7	NED	ATGTACAGGTTCCAGTGTCCG	CCAGTTCATACAACCTTGAGTTGG	119-143
ADL0259	9	VIC	CTCATTGCAGAGGAAGTTCT	GTAATGGAGGATGCTCAGGT	107-129
GCT0016	9	NED	TCCAAGGTTCTCCAGTTC	GGCATAAGGATAGCAACAG	109-125
**MCW0228**	**10**	**PET**	**GATCTCTGCATTACAAGCATG**	**TTGCTGACCTGCTCATGCAAG**	**221-239**
MCW0104	13	FAM	TAGCACAACTCAAGCTGTGAG	AGACTTGCACAGCTGTGTACC	189-225
ROS0083	13	VIC	CATTACAGCTCAGTGTTGGCA	TTGCAAGTGCTCTCCCATC	109-129
**MCW0213**	**13**	**NED**	**GACAAGTCAACAACTTGCCAG**	**CTGTTCACTTTAAGGACATGG**	**288-316**
MCW0123	14	FAM	CCACTAGAAAAGAACATCCTC	GGCTGATGTAAGAAGGGATGA	79-89
ADL0293	17	PET	GTAATCTAGAAACCCCATCT	ACATACCGCAGTCTTTGTTC	105-119
MCW0330	17	VIC	TGGACCTCATCAGTCTGACAG	AATGTTCTCATAGAGTTCCTGC	254-286
ADL0304	18	FAM	GGGGAGGAACTCTGGAAATG	CCTCATGCTTCGTGCTTTTT	137-159
**LEI0074**	**26**	**VIC**	**GACCTGGTCCTGACATGGGTG**	**GTTTGCTGATTAGCCATCGCG**	**224-240**
**LEI0135**	**28**	**NED**	**CACAATGAAGGATGAATAGTGC**	**AATTCACAGTTACACCTGAGG**	**131-142**

*Bold is selected 12 MS marker combination.

The Polymerase Chain Reaction (PCR) was performed in total volume of 20 μL, 50 ng of genomic DNA, 10 pmol of fluorescent dye (FAM, VIC, NED, PET) labeled modified forward primer and normal reverse primer (Applied Biosystems, USA), 2.5 mM of each dNTPs (GeNet Bio, Korea), 10 X reaction buffer (GeNet Bio, Korea), 2.5 unit of prime Taq DNA polymerase (GeNet Bio, Korea). The PCR was performed in an initial denaturation at 95°C for 10 min followed by 35 cycles of 30 sec of denaturation at 95°C, 30 sec of annealing at 60°C, 30 sec of extension at 72°C and final extension at 72°C for 10 min using My-Genie 96 Thermal Cycler (Bioneer, Korea). The PCR products were initially electrophoresis on 3% agarose gel with ethidium bromide (EtBr) and confirmed whether they gave single PCR DNA band under the UV light. When the bands were clearly appeared, further genotyping was performed. For the microsatellite genotyping, more than 20 times diluted PCR products were used. The genotyping reaction contained 1 μL of diluted PCR products, 10 μL of Hi-Di™ Formamide (Applied Biosystems, USA) and 0.1 μL of GeneScan™-500 LIZ™ size standard marker (Applied Biosystems, USA). After dilution, genotyping reaction mixture was denatured for 2 min at 95°C and fragment analysis was performed using capillary array in Genetic analyzer 3130xl (Applied Biosystems, USA). The MS genotypes were identified using GeneMapper ver.3.7 (Applied Biosystems, USA).

### Genetic diversity and population structure analysis

The genotyping data were used to estimate mean number of allele (Na), Hexp, observed heterozygosity (Hobs) and PIC using Cervus 3.0 program [15]. The expected probability of identity values among genotypes of random individuals (PI), random half sibs (PI_*half-sibs*_) and random sibs (PI_*sibs*_) were calculated using API-CALC (Average Probability of Identity-Calculate) ver 1.0 [16]. Moreover, we used both model-based and non model-based methods to describe the diversity between pre-defined genetic clusters. We used STRUCTURE software v. 2.3 [17] for model-based and DAPC (Discriminant Analysis of Principal Components) program implemented in adegenet R-package [18-20] for non model-based method. STRUCTURE results were also used to assess population structure of Korean breeds.

DAPC analysis was preceded by the execution of the *K*-means clustering algorithm implemented in adegenet to identify an optimal number of genetic clusters to describe the data. For this purpose, we ran *K*-means sequentially with increasing number of clusters. Different clustering solutions are compared using Bayesian Information Criterion (BIC). The optimal number of groups matches the lowest BIC value. Also, genetic distance [21] values were computed by PowerMarker ver 3.25 [22]. We used the number of loci which differ between two individuals as a measure of the genetic distance between individuals. This was computed using the R package ape [23]. A neighbor-joining tree was then constructed based on the resulting distance matrix using the same package. Genetic distance between breeds was computed using Reynolds genetic distance (which is an allele frequency-dependent distance). NeighborNet graph was computed using splitstree software [24].

## Results and discussion

### Polymorphisms of microsatellite markers

A total of 215 alleles were detected from 30 MS markers in WM2, ranging from 5 to 13 alleles with an average of 7.17 alleles per locus. The Hexp values ranged from 0.474 for ADL0304 to 0.841 for MCW0264. The Hobs values varied from 0.151 for GCT0016 to 0.885 for ADL0159, with an average of 0.741 per locus in the WM2 population. The obtained average PIC value per locus in WM2 was 0.682 and varied from 0.443 for ADL0304 to 0.819 for MCW0264 (Table 2). These markers were also polymorphic in other chicken populations. The lowest value of Na, Hexp, and PIC was calculated in the RIR population (Additional file 1: Table S1). Seo et al. [14] reported the classification of five different lines of KNC using 15 MS markers and determined the mean Na, Hexp, Hobs, and PIC values of 8.4, 0.802, 0.709, and 0.771, respectively. With the exception of the Hobs values, the values in the current study were lower than those reported by Seo et al. [14]. Of the 30 selected markers, six (GCT0016, MCW0029, MCW0063, MCW0087, MCW0264, and MCW0104) were used in the study by Seo et al. [14]. Furthermore, four markers (LEI0135, MCW0111, MCW0145, and MCW0330) were investigated by Suh et al. [25] for the discrimination of four different breeds, including the WM1 population. Our marker combination is more polymorphic than that in the study by Suh et al. [25]. In addition, of the 30 markers in this study, six were among the ISAG recommended markers (ADL0268, LEI0094, MCW0104, MCW0111, MCW0123, and MCW0330). These results indicated that highly polymorphic MS markers were commonly used in diverse populations.

**Table 2:** The heterozygosity (Hobs and Hexp) and polymorphism information content (PIC) values using 30 MS markers in Woorimatdag version 2 (WM2) chicken population*

**Locus**	**No. of allele**	**HObs**	**HExp**	**PIC**
ADL0268	5	0.783	0.742	0.703
MCW0111	5	0.789	0.695	0.654
MCW0145	7	0.754	0.692	0.655
**MCW0063**	**7**	**0.775**	**0.783**	**0.746**
**MCW0087**	**9**	**0.833**	**0.785**	**0.751**
**LEI0141**	**7**	**0.832**	**0.797**	**0.767**
**MCW0039**	**7**	**0.738**	**0.765**	**0.726**
**MCW0264**	**8**	**0.877**	**0.841**	**0.819**
MCW0288	6	0.608	0.631	0.578
MCW0127	6	0.742	0.679	0.627
MCW0040	6	0.813	0.695	0.656
**ADL0317**	**7**	**0.819**	**0.775**	**0.739**
LEI0094	7	0.845	0.716	0.665
ADL0292	7	0.774	0.733	0.684
MCW0029	13	0.805	0.815	0.790
**ROS0013**	**8**	**0.871**	**0.771**	**0.734**
**ADL0159**	**7**	**0.885**	**0.789**	**0.757**
ROS0019	8	0.766	0.643	0.581
ADL0259	6	0.674	0.657	0.620
GCT0016	9	0.151	0.804	0.773
**MCW0228**	**6**	**0.821**	**0.773**	**0.740**
MCW0104	11	0.743	0.813	0.786
ROS0083	8	0.818	0.749	0.707
**MCW0213**	**11**	**0.807**	**0.798**	**0.771**
MCW0123	5	0.707	0.687	0.627
ADL0293	7	0.701	0.554	0.522
MCW0330	6	0.484	0.643	0.592
ADL0304	5	0.513	0.474	0.443
**LEI0074**	**6**	**0.85**	**0.745**	**0.704**
**LEI0135**	**5**	**0.659**	**0.596**	**0.544**
**Mean**	**7.17**	**0.741**	**0.721**	**0.682**

*Bold is selected 12 MS marker combination.

Bostein et al. [26] reported that markers with PIC values >0.5 and Hexp values >0.6 have a high polymorphic information content and are sufficient for breed discrimination. Thus, our results confirmed that all markers, with the exception of ADL0304, have high polymorphic information and suitable allele frequencies and polymorphisms, and can be used to discriminate the WM2 population.

### Discrimination of WM2 population

**Figure 1:**
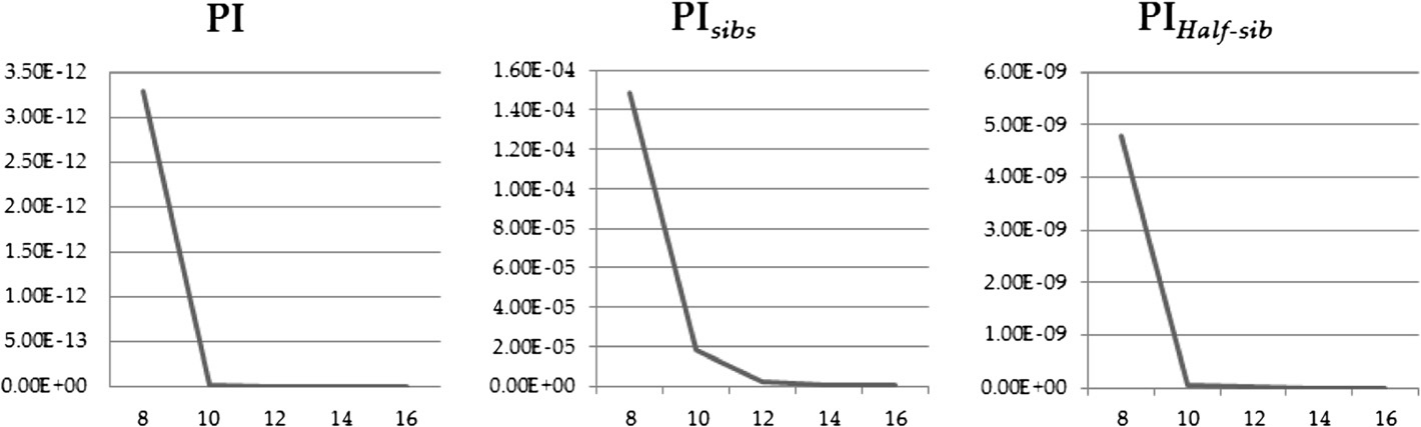
**The expected probability of identity values among genotypes of random individuals (** ***PI*** **), random half-sib** ***(PI***_***half-sibs***_ **) and random sibs (** ***PI***_***sibs***_ **) were suggested markers for discrimination of chicken lines.**

While all 30 selected markers had the ability to discriminate the WM2 population from other populations, the best minimum number of markers was required from an economic point of view. For this reason, 12 MS markers were selected for the best minimum MS marker combination based on the highest Hexp and PIC values. Using this combination of 12 MS markers, the calculated *PI*, *PI*_*half-sibs*_, and *PI*_*sibs*_ values were 3.23E-33, 5.03E-22, and 8.61E-08, respectively. A previous study, using 12 markers, reported *PI*, *PI*_*half-sibs,*_ and *PI*_*sibs*_ values of 7.98E-29, 2.28E-20, and 1.25E-8, respectively, for the discrimination of 5 lines of purebred KNCs [14]. These results suggest that the selected 12 markers have high polymorphism and are effective in discriminating the WM2 population from other populations (Figure 1).

### Genetic distance among WM2 and other populations

To establish genetic relationships among WM2 and the other populations, genetic distances were calculated using the alleles from the 12 selected MS markers. Nei et al. [21]’s genetic distance was calculated between WM2 and the other populations using a pairwise co-ancestry matrix according to the allele frequencies (Table 3). The lowest genetic distance (0.1375) was observed between the WM1 and CoL populations. The genetic distance between O chicken population and WM2 was the highest (0.791), followed by RIR and WM2 (0.788) (Table 3). Similarly, Suh et al. [25] reported the lowest genetic distance (0.092) between the WM1 and Hh populations and the highest genetic distance (0.690) between the RIR and White Leghorn breeds. This indicated that the WM1 and Hh populations originated from the same breed/ancestor for constructing the populations. Furthermore, according to the genetic distance values, our marker combination has a stronger discriminating power than that in the findings by Suh et al. [25]. On the other hand, close genetic distances of the WM1 population with the CoL, Hh, and RIR populations (0.1375, 0.2453, and 0.2478, respectively) were observed. These results support the findings by Suh et al. [25] that the WM1 and Hh populations have the same founder breeds as their genetic distances are close (Table 3). The WM2 population, however, has a genetic distance >0.690 indicating that different crossing combinations were applied between the WM2 and WM1 populations.

**Table 3:** Matrix of genetic distances among nine populations*

	**Bb**	**CoL**	**CoR**	**Hh**	**Hn**	**O**	**RIR**	**Wm1**	**Wm2**
**Bb**	-								
**CoL**	0.3267	-							
**CoR**	0.3251	0.3906	-						
**Hh**	0.2715	0.3114	0.3805	-					
**Hn**	0.4189	0.4090	0.4738	0.4460	-				
**O**	0.4587	0.4027	0.4543	0.4972	0.5287	-			
**RIR**	0.4371	0.4702	0.5382	0.3494	0.5492	0.6302	-		
**Wm1**	0.2791	0.1375	0.3365	0.2453	0.3667	0.4341	0.2478	-	
**Wm2**	**0.7102**	**0.7225**	**0.7095**	**0.7121**	**0.7618**	**0.7914**	**0.7878**	**0.6904**	-

*WM1 (Woorimatdag version 1), WM2 (Woorimatdag version 2), Hh (Hanhyup-3), Hn (Hyunin), RIR (Rhode Island Red), CoL (Cornish black), CoR (Cornish red), O (Ogye) and Br (Broiler).

### Phylogenetic and structure analysis of nine populations

**Figure 2:**
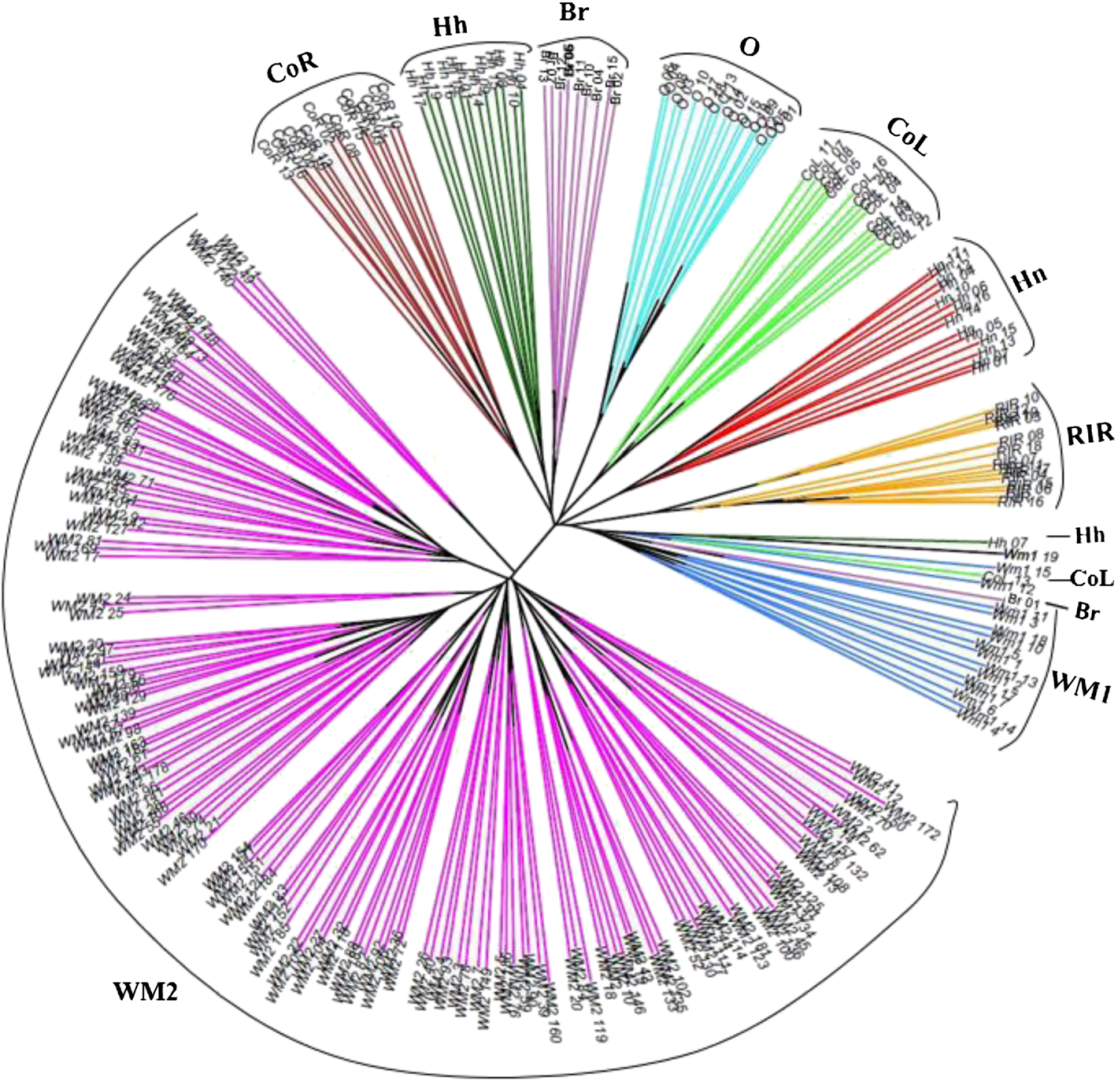
**Phylogenetic analysis for each individual from nine populations using Reynolds genetic distance.** The color codes are indicated different populations. The population acronyms are as follows: WM1 (Woorimatdag version 1), WM2 (Woorimatdag version 2), Hh (Hanhyup-3), Hn (Hyunin), RIR (Rhode Island Red), CoL (Cornish black), CoR (Cornish red), O (Ogye) and Br (Broiler).

Based on Nei’s equations [21], an unrooted neighbor-joining (NJ) phylogenetic tree was constructed for 263 animals from nine chicken populations using 12 MS marker variations (Figure 2). In our individual phylogenetic analysis, the WM2 population was identified as a distinct population from other populations. O chicken population was also well separated from other populations and a mixture clade contained the WM1, Hh, CoL, and Br populations.

**Figure 3:**
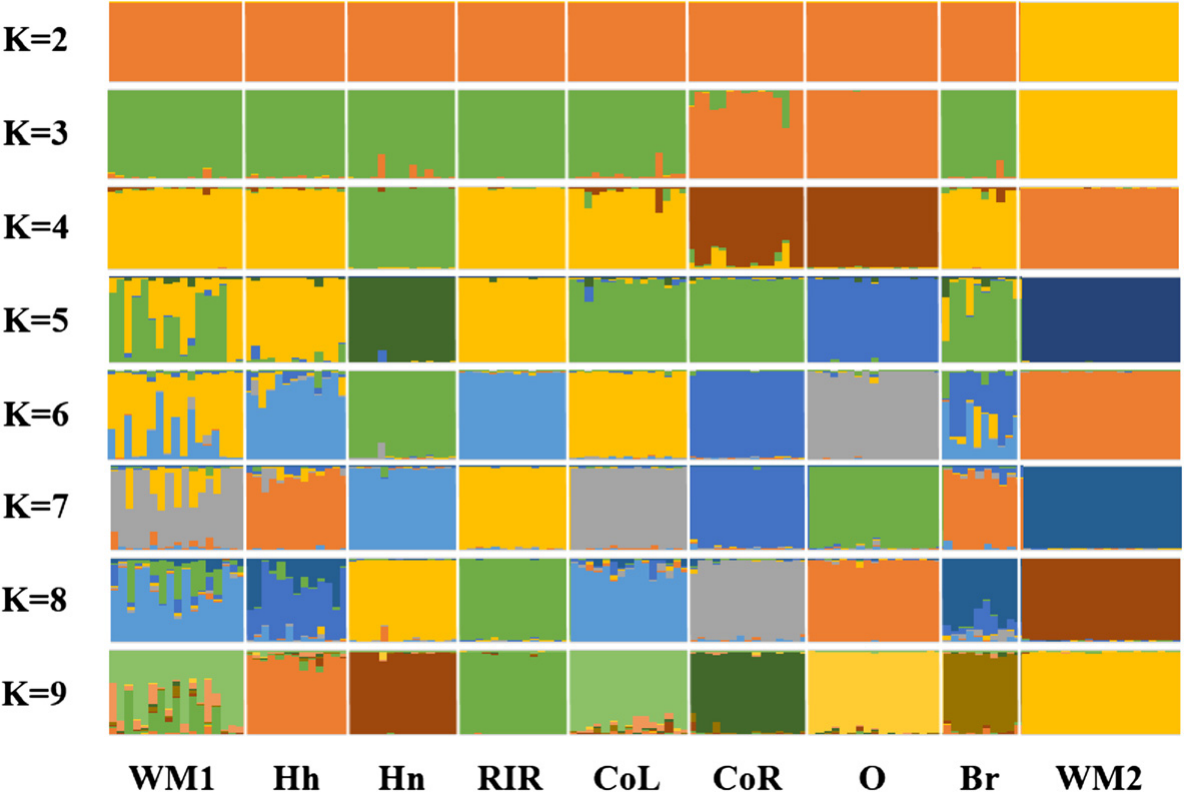
**Structure analysis using twelve MS markers from nine populations.** The population acronyms are as follows: WM1 (Woorimatdag version 1), WM2 (Woorimatdag version 2), Hh (Hanhyup-3), Hn (Hyunin), RIR (Rhode Island Red), CoL (Cornish black), CoR (Cornish red), O (Ogye) and Br (Broiler).

The genetic structure of nine native chicken populations using microsatellite marker genotypes was investigated based on population clustering (Figure 3). The purpose of structure analysis, performed using a Bayesian approach based on the marker genotypes, was to delineate clusters of individuals [27]. Using 12 MS markers and a K value of 2, WM2 was fully separated from the other populations. This result was also observed in the individual phylogenetic and discriminant analyses. Based on these results, the combination of 12 MS markers could discriminate WM2 from the other populations. Furthermore, with a K value of 9, most populations classified well with different groups. WM1, however, was found to be a mixture population, a finding consistent with the results obtained from the phylogenetic and DAPC analyses. The discriminant analysis confirmed the WM2 populations were distinct from the other populations.

**Figure 4:**
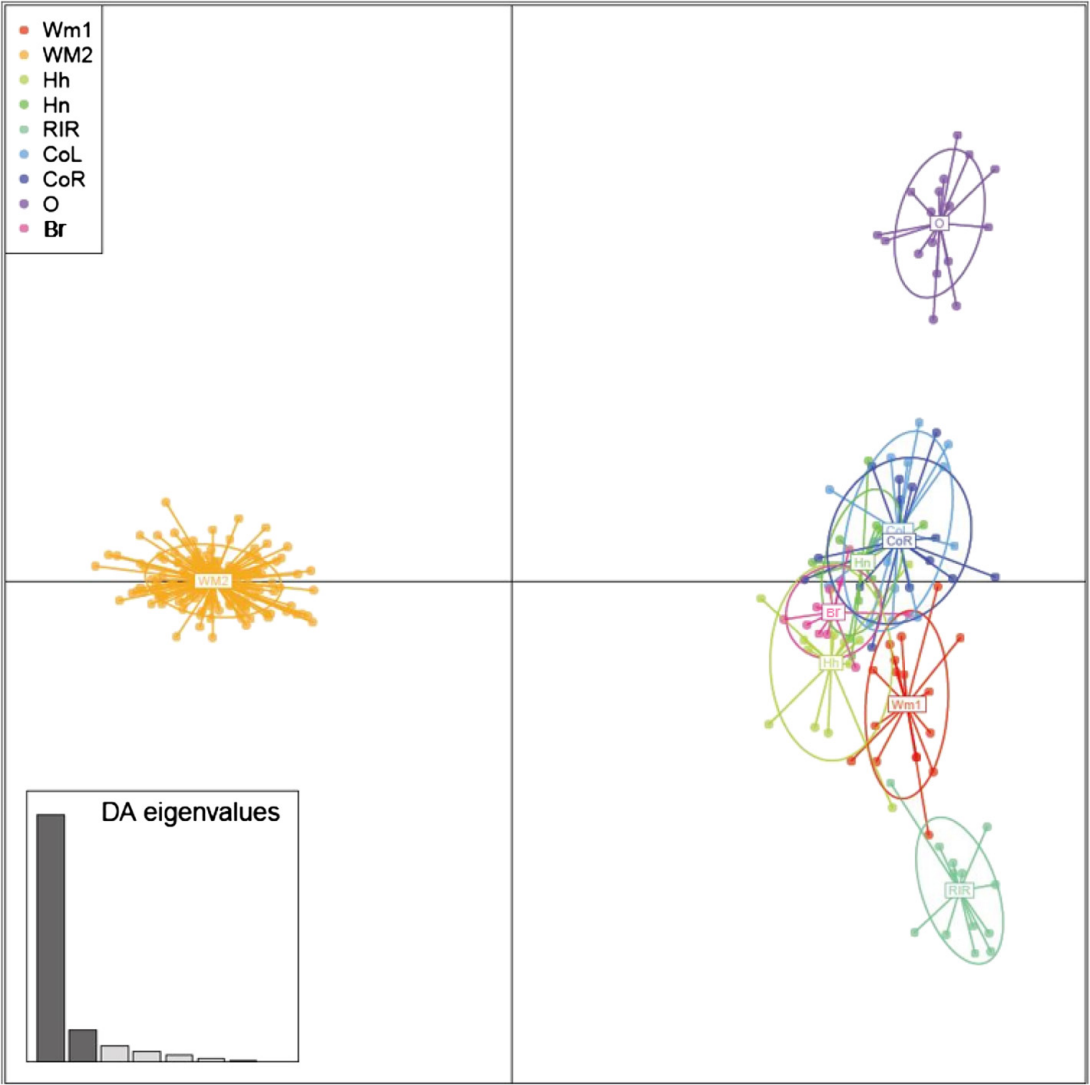
**Scatter plot of DAPC analysis of the nine populations using adegent R package.** The population acronyms are as follows: WM1 (Woorimatdag version 1), WM2 (Woorimatdag version 2), Hh (Hanhyup-3), Hn (Hyunin), RIR (Rhode Island Red), CoL (Cornish black), CoR (Cornish red), O (Ogye) and Br (Broiler).

The assignment of the individuals from the 9 populations was 9 clusters (group), represent genetic groups and they were inferred using *K*-means algorithm implemented in the R package adegenet (Figure 4). Furthermore, individuals (represented by dots) were plotted according to their coordinates on the first two principal components. The populations were represented as inertia ellipses, which characterize the dispersion of each population around its center of gravity. Bar graph insets indicate the amount of variance determined by the two discriminant values used for plotting. WM2 is clearly separated from the other populations, a result supported by the phylogenetic analysis using 12 selected MS markers. The 12 selected MS markers were also used for the separation of the O chicken and RIR groups.

## Conclusions

Since our 12 MS marker combination can effectively discriminate WM2, they can be used for breed identification. Moreover, to the best of our knowledge, this is the first study demonstrating the discrimination of the commercial KNC population, and the results presented here may be applied in the commercial market.

## Additional file

Additional file 1: Table S1. The calculated number of alleles (k), observed heterozygosity (HObs), expected heterozygosity (HExp), and polymorphic information content (PIC) values in eight chicken populations.

## Abbreviations

BIC: Bayesian Information Criterion
Br: Broiler
CoL: Cornish Black
CoR: Cornish Red
DAPC: Discriminant Analysis of Principal Components
EtBr: Ethidium Bromide
Hexp: Expected heterozygosity
Hh: Hanhyup-3
Hobs: Observed heterozygosity
Hn: Hyunin
ISAG: International Society for Animal Genetics
KNC: Korean native chicken
MS: Microsatellite
Na: Number of allele
NIAS: National Institute of Animal Science
NJ: Neighbor-joining
O: Korean Ogye
PCR: Polymerase Chain Reaction
PI: Probability of identity
PIC: Polymorphic Information content
QTL: Quantitative Trait Locus
RIR: Rhode Island Red
SSR: Simple-Sequence Repeat
WM1: Woorimatdag version 1
WM2: Woorimatdag verson 2
